# Thermophysical Behavior of Carbonated Aqueous Solutions Containing Monoethanolamine and Degradation Products

**DOI:** 10.1021/acs.jced.4c00077

**Published:** 2024-05-09

**Authors:** Clàudia Rosa Hernández Narciso, Cristina G. Martínez, Brendan O’Connell, Sabrina Belén Rodriguez Reartes, Fèlix Llovell, J. P. Martin Trusler, Kyra L. Sedransk Campbell

**Affiliations:** † Department of Chemical Engineering and Materials Science, IQS, Universitat Ramon Llull, Via Augusta 390, 08017 Barcelona, Spain; ‡ Department of Chemical Engineering, Imperial College London, South Kensington Campus, London SW7 2AZ, United Kingdom; § Department of Chemical Engineering, ETSEQ, Universitat Rovira i Virgili, Av Països Catalans 26, 43007 Tarragona, Spain; ∥ Planta Piloto de Ingeniería Química (PLAPIQUI-UNS-CONICET), Camino “La Carrindanga” km 7, Bahía Blanca 8000, Argentina; ⊥ Department of Chemical and Biological Engineering, The University of Sheffield, Sheffield S1 4AA, United Kingdom

## Abstract

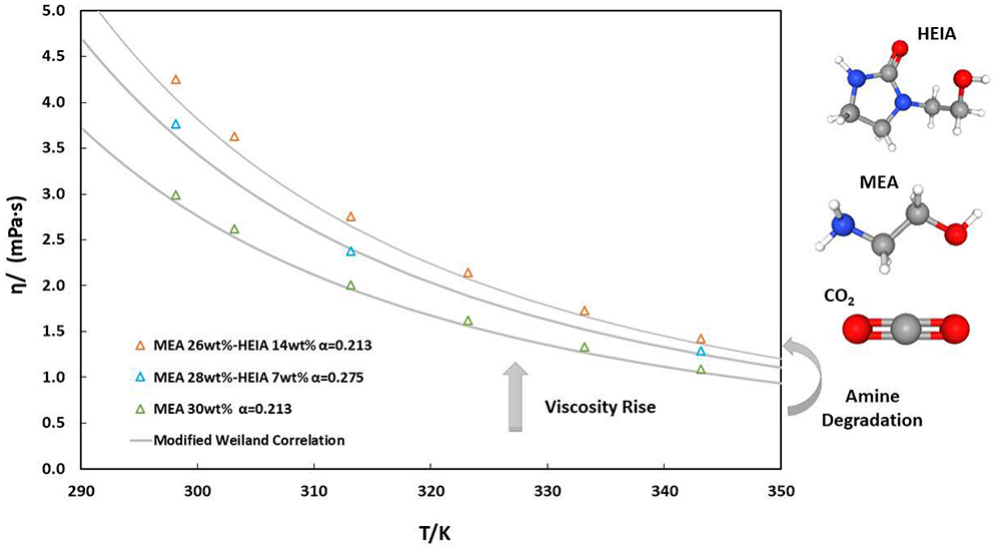

The impact of the degradation of monoethanolamine (MEA) on the physicochemical properties of the solvent is experimentally characterized. Based on the identification of three main degradation products of MEA: oxazolidine-2-one (OZD), N-(2-hydroxyethyl)ethylenediamine (HEEDA), and 1-(2-hydroxyethyl)-2-imidazolidinone (HEIA), new measurements for the density, surface tension, and viscosity of partially carbonated solutions containing water, MEA and those products were conducted at different MEA/degradation product molar ratios. The experiments covered a temperature range from 298.15 to 353.15 K at atmospheric pressure. The more stable and impactful degradation product, HEIA, was analyzed separately to determine its vapor pressure, as well as the density and viscosity of aqueous solutions with HEIA mass fractions of 100, 75, 50, and 25% in the same temperature range. The reported data demonstrate the difference in the performance of aqueous MEA solutions containing degradation products as compared to a fresh solution. This aspect is crucial for understanding the impact and effectiveness of postcombustion CO_2_ capture using aqueous amine systems in an industrial setting.

## 1 Introduction

Carbon dioxide (CO_2_) emitted by anthropogenic sources is a significant driver of the observed changes in the Earth’s climate. Therefore, the development of efficient and cost-effective CO_2_ capture techniques is considered one of the highest priorities in the field of Carbon Capture and Storage (CCS) to mitigate its effects. In particular, Post-Combustion Capture (PCC) followed by geological storage holds the promise of significant CO_2_-emission reductions from existing power stations and industrial processes.^1^ It is widely recognized that the advanced technological stage of PCC offers significant advantages for widespread large-scale implementation. Moreover, the ability to retrofit it to existing point sources enhances its potential.^2^ Aqueous amine-based chemical absorption is the dominant process employed in PCC.^3^ In the absorption column, which operates at moderate temperatures and ambient pressure, the gas stream enriched with CO_2_ undergoes a reaction with the amine species present in the solution. This reaction is reversible when the temperature is elevated in the desorber, allowing the selective release of CO_2_ at the top of the column. Once the solvent is, theoretically, free from CO_2_ (although a low CO_2_ concentration typically remains), it can be pumped back to the absorber in a steady-flow process.^2,4,5^

Monoethanolamine (MEA) is considered the “benchmark” amine due to its historical use, which in turn makes data on its properties and performance characteristics widely available. It remains commercially popular due to its appealingly high cyclic capacity, fast kinetics at low CO_2_ partial pressure, low viscosity, high water solubility, low price, etc. Unsurprisingly, it also has disadvantages. For instance, the energy requirements for PCC, with aqueous MEA as the solvent, amounts to 27% of the gross capacity of a power plant. This energy requirement is mainly for solvent regeneration and circulation, compression of CO_2_, and fan power. In addition, MEA makeup requirements contribute about 10% to the cost, mostly caused by solvent degradation.^6^

Amine degradation is an irreversible process and three different pathways can be distinguished.^3,7−8^ First, an oxidative degradation is expected to happen in the liquid holdup at the bottom of the absorber (at 313–343 K) and in the heat exchanger (at 373–418 K) in the presence of oxygen and other oxidative contaminants (NO_X_ and SO_X_), as well as with an iron catalyzer.^7,9^ The products are typically oxidized fragments of the solvent such as acetates, formates, glycolates, and ammonia. This type of degradation is a problem for CO_2_ capture from a flue gas stream where the O_2_ concentration is typically greater than 3%.

Second, purely thermal degradation may also occur, although it is typically not considered for CO_2_ capture with amine solvents, given that it only takes place when the temperature is higher than 473.15 K.^9,10^

Finally, carbamate polymerization typically occurs in the stripper at high temperatures (between 373.15 and 473.15 K) and in the presence of CO_2_, producing high molecular weight polymers when reaching the highest temperatures in the equipment. However, only primary and secondary alkanolamines go through this degradation mechanism, since they can form carbamate molecules that react with CO_2_ to form oxazolidone (OZD).^11,12^ The temperature is identified as a crucial parameter for regulating the degradation process in chemical absorption. While operating at lower temperatures in the stripper decreases the degradation rate and minimizes solvent makeup, an excessively sharp reduction in temperature hinders the solvent regeneration process, leading to increased costs. Therefore, achieving an optimal balance between solvent regeneration costs and solvent makeup is essential to optimize the stripping process.^7−9^

**Figure 1:**
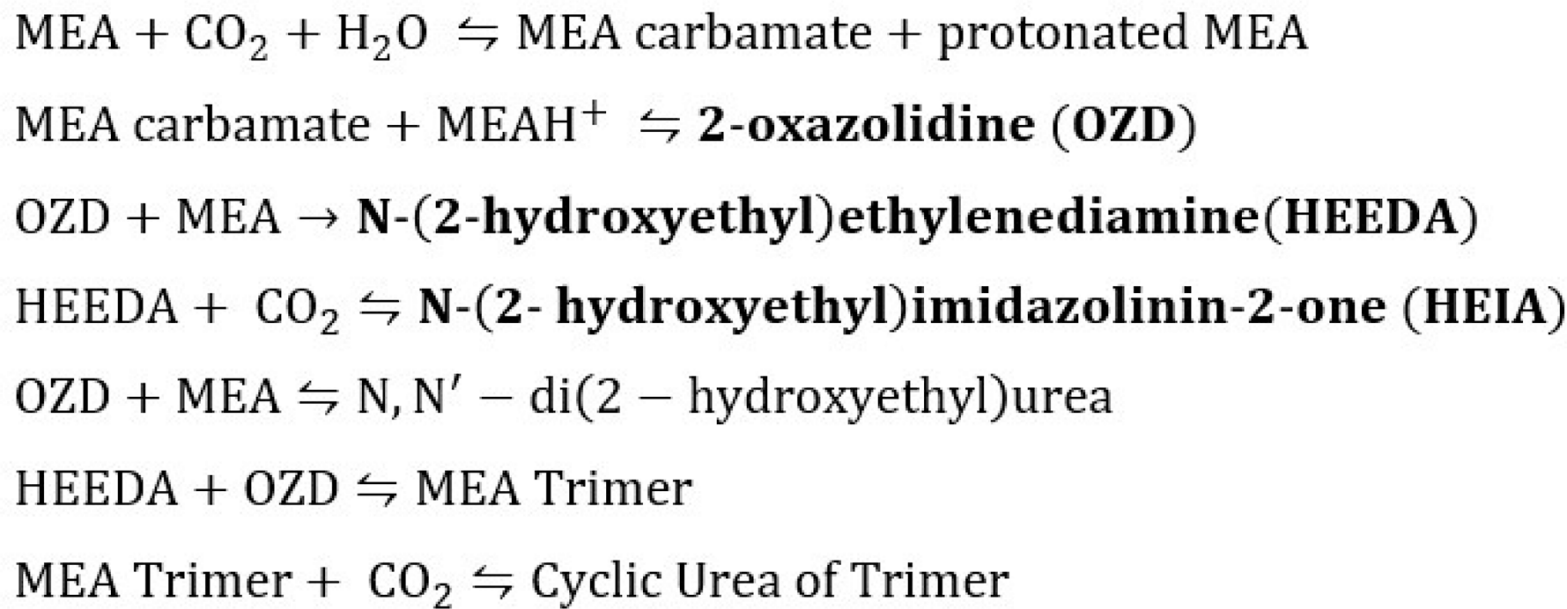
MEA thermal degradation pathway in the presence of CO_2_.^9^

Focusing on the latter, a mechanism for carbamate polymerization degradation of MEA was proposed by Polderman et al. for the first time in 1956,^12^ and further refined in subsequent research.^9^ A summary of the reactions is given in Figure 1.

As can be observed, the degradation starts when the MEA carbamate, formed in the CO_2_ capture mechanism, reacts with a protonated MEA molecule to produce 2-oxazolidone (OZD). Subsequently, OZD reacts with MEA to form N-(2-hydroxyethyl) ethylenediamine) (HEEDA) in what is known to be an irreversible reaction.^9,13^ However, this byproduct is susceptible to thermal degradation and becomes either 1-(2-hydroxyethyl)-2-imidazolidinone (HEIA) or a MEA trimer.^14,15^ The formation of HEIA occurs as a result of the reaction between HEEDA and CO_2_, while the formation of an MEA trimer requires a reaction between HEEDA and OZD. The MEA trimer has been observed to polymerize further and/or undergo intramolecular cyclization reactions.^9,11,12,16^ In any case, the impact of HEIA is the most critical, being the largest degradation product found in the solution.^9,17,18^ Reactions of HEIA to form further polymeric products have not been found. Consequently, according to this mechanistic pathway, an increasing concentration of HEIA is expected over time. This fact has been experimentally confirmed, further highlighting its relative stability.^9,19,20^

To date, most research has focused on understanding the chemical pathways involving the commonly observed byproducts. The accumulation of these compounds is related to corrosion, fouling, increased viscosity, and foaming,^3,21,22^ reducing the process efficiency and, ultimately, causing financial losses. The viscosity, along with other physical properties of aqueous-MEA solutions, such as the density and the surface tension, is intimately related to other noted problems, including formation and stability, mass-transfer resistance, and increased costs for solvent pumping. As a consequence, it is of key importance to unveil how these physicochemical properties are affected by the presence of degradation products.

While the density, surface tension, and viscosity of binary and ternary solutions of aqueous amines over a large range of composition and temperature have been widely studied in the literature,^23−32^ there is virtually no data available for these properties in solutions including the addition of relevant impurities, particularly degradation products, as only reaction rates of oxidative and thermal degradation have been studied.^9,22,33^ In particular, Braakhuis et al. have recently developed a kinetic model to predict the thermal degradation rate of MEA and the formation rates of HEEDA and HEIA as a function of time, temperature, and loading. The degradation model confirms that the formation of HEIA is a function of the concentration of HEEDA and CO_2_ and that, at normal stripper temperatures (393.15 K) is limited, whereas it increases at higher temperatures (408.15 K).^22^ Therefore, the goal of this work is to study the influence of three critical degradation compounds, HEIA, OZD, and HEEDA, on the physicochemical properties of an aqueous MEA solution at typical CO_2_ capture conditions. In particular, the density, the viscosity, and the surface tension are evaluated at different temperature conditions. Departing from a common aqueous solution of MEA of molality 7 mol kg^–1^ (corresponding to a MEA mass fraction of 30%) as a benchmark, additional solutions, where part of the MEA is substituted by HEIA, OZD, or HEEDA, with and without CO_2_ loadings, have been measured. Different degrees of degradation have been checked so as to fully characterize their physicochemical behavior. In this way, the impact of common and unavoidable degradation products on the properties of an amine solution has been quantified for the temperature range of the absorption process, 293.15–353.15 K.

## 2 Materials and Experimental Methods

### 2.1 Sample Preparation

The chemicals used in this study are detailed in Table 1. HEIA (normal melting temperature of 327 K) was purchased as an aqueous solution. The pure compound was obtained by dehydrating the solution in a Buchi R3 Rotovap vacuum rotary evaporator operating at a temperature of 368.15 K and at pressure below 5 kPa. The mass fraction of HEIA in the supplied solution was gravimetrically determined by drying a small sample of known initial mass in an oven to constant mass. The tested solutions were prepared gravimetrically with an expanded uncertainty in mass fraction of 0.0005 (*k* = 2) starting from either pure compounds or, in the case of HEIA, the original aqueous solution. Stock solutions were stored in sealed glass bottles to prevent absorption of atmospheric CO_2_.

**Table 1:** Description of Chemical Samples^a^

Chemical name	CAS number	Abbreviation	Supplier	Purity as supplied	Additional purification
Carbon dioxide	124-38-9		BOC	*x* = 99.995%	None
Water	7732-18-5		N/A^b^	ρe = 25 MΩ cm	Degassed
Ethanolamine	141-43-5	MEA	Sigma-Aldrich	*w* = 99.0%	None
2-Oxazolidone	497-25-6	OZD	VWR Int.	*w* = 99.0%	None
N-(2-Hydroxyethyl) ethylenediamine	111-41-1	HEEDA	Sigma-Aldrich	*w* = 99.0%	None
1-(2-Hydroxyethyl)-2-imidazolidinone	3699-54-5	HEIA	Sigma-Aldrich	*w* = 75.0%^c^	Dehydration

a *x* is mole fraction, *w* is mass fraction, and ρ_e_ denotes electrical resistivity at *T* = 298.15 K.

b Obtained from a Millipore Direct Q-UV water purification system.

c Purity of dried HEIA is *w* ≈ 95%.

Carbonated solutions were prepared by bubbling CO_2_ through samples of approximately 250 mL of the degassed stock solution at a flow rate of approximately 80 mL min^–1^ and at ambient temperature of (294 ± 1) K for 4 h. During the saturation period, the pH solution was periodically monitored to establish when saturation was achieved. “Half-loaded” solutions were prepared by dilution with unloaded stock solution. Finally, the actual CO_2_ loading was determined before each measurement by the BaCl_2_ methods and acid–base titration.^34,35^

The compositions of the stock solutions used in this study are specified in Table 2, along with the experimental temperature range and the approximate degree of CO_2_ saturation considered. Additionally, the density, enthalpy of vaporization, and vapor pressure of pure HEIA were determined (see Table 4 and the Supporting Information for further details).

**Table 2:** Experimental Matrix for Studied Solutions^a^

Comp 3	10^2^ *w*_2_	10^2^ *w*_3_	*b*_2_/*m*^0^	*b*_3_/*m*^0^	Properties	*T*_min_ (K)	*T*_max_ (K)	α/α_sat_
–	29.80	0.00	6.95	0.00	ρ, η, σ	298.15	343.15	0, 0.5, 1.0
HEIA	0.00	24.10	0.00	2.44	ρ, η	298.15	333.15	0
HEIA	0.00	48.80	0.00	7.32	ρ, η	298.15	353.15	0
HEIA	0.00	72.80	0.00	20.59	ρ, η	298.15	353.15	0
HEIA	28.01	6.64	7.02	0.78	ρ, η, σ	298.15	343.15	0, 0.5, 1.0
HEIA	25.87	13.78	7.02	1.75	ρ, η, σ	298.15	343.15	0, 0.5, 1.0
HEEDA	28.00	4.00	6.74	0.56	ρ, η, σ	298.15	343.15	0, 0.5, 1.0
OZD	25.00	7.00	6.74	0.68	ρ, η, σ	298.15	343.15	0, 0.5, 1.0

a *w*_*i*_ denotes mass fraction of component *i* in the stock solution prior to absorption of CO_2_, *b*_*i*_ denotes the corresponding molality with water as the solvent, *m*_0_ = 1 mol kg^–1^, *T*_min_ and *T*_max_ are the minimum and maximum experimental temperatures, and α/α_sat_ is the approximate degree of CO_2_ saturation at the solution preparation temperature of (294 ± 1) K. Component 2 is MEA while component 3 is identified in the table.

### 2.2 Density Measurements

The density of the aqueous carbonated solutions was measured with an Anton Paar DMA 5000 M vibrating-tube densimeter that was calibrated at *T* = 293.15 K with ambient air and pure water.^36^ Temperature was determined with a platinum resistance thermometer integrated within the DMA 5000 M densimeter with an expanded uncertainty of 0.02 K (*k* = 2). The expanded uncertainty of the density measurements is provided within the measurement results tables. Measurements were generally made at temperatures between 298.15 and 353.15 K. During the study of carbonated solutions at high temperatures, degassing was occasionally observed, leading to the abandonment of the measurement in such cases.

Density measurements on pure HEIA were performed using an external high-temperature high-pressure measurement cell, an Anton Paar DMA HP densimeter, connected to the DMA 5000M. This external cell permitted measurements at higher temperatures. The HEIA was melted in a glass beaker on a hot plate and drawn into the densimeter measurement cell with a syringe. Measurements on the pure substance were performed at temperatures between 333.15 and 363.15 K, since HEIA is solid below 323.15 K. The expanded uncertainty of HEIA density measurements carried out with this apparatus is typically 0.0001 g cm^–3^, as determined by careful calibration and validation measurements.^37^ However, in the present case, the repeatability was lower, and an expanded uncertainty of 0.002 g cm^–3^ is estimated.

### 2.3 Viscosity Measurements

Kinematic viscosity was measured with a certified Ostwald-type capillarity viscometer (PSL Rheotek), partially submerged in an oil bath thermostat (Julabo 18 V). Temperature was measured with a calibrated secondary-standard platinum resistance thermometer (Fluke model 5615) with an expanded uncertainty of 0.01 K (*k* = 2). The capillary sizes used and the corresponding recommended kinematic viscosity ranges were O (0.3 to 1 mm^2^ s^–1^), A (0.9 to 3 mm^2^ s^–1^), B (2 to 10 mm^2^ s^–1^), and C (6 to 30 mm^2^ s^–1^). Measurements were carried out at temperatures between 298.15 and 353.15 K in steps of 10 K, discarding any measurement in which degassing was observed. The flow time was measured with an electronic stopwatch, repeating all measurements three times. The estimated expanded relative uncertainty of the viscosity is provided within the measurement results tables.

### 2.4 Surface Tension Measurements

The surface tension was measured by means of the reverse pendant drop method (or sessile drop method) wherein a bubble of CO_2_-free air was formed at the open end of a vertical capillary inserted into the solution. For this purpose, a Ramé-Hart Advanced goniometer was employed with a 0.72 mm O.D. inverted needle (Ramé-Hart) attached to a micro syringe assembly and lowered into the liquid. The sample was contained by a 45 mL quartz cuvette mounted within a thermostatic chamber. Images of the drop were captured with the goniometer’s camera system and analyzed with the DROPimage Advanced v2.6 software (Ramé-Hart) to determine the surface tension by solution of the Young–Laplace equation. The goniometer’s imaging system was calibrated using a spherical calibration tool and validated by measuring the surface tension of pure water, obtaining results that agreed to within ±0.4 mN m^–1^ with the IAPWS recommended value of 72.0 mN m^–1^ at *T* = 298.15 K.^38^ The sample temperature was measured with an expanded uncertainty of 1.5 K (*k* = 2) using a K-type thermocouple immersed in the liquid. Solution densities (required to compute the surface tension from the drop image) were taken from the present work. The measurements were made at temperatures between 298.15 and 333.15 K and the expanded relative uncertainty, based on the mean of four replicated measurements, was 1.2 mN/m.

### 2.5 Vapor Pressure Measurements

The vapor pressure of pure HEIA was measured using the electrically heated glass ebulliometer described by Deschamps et al.^39^ This apparatus was designed for measurements of low vapor pressures in the range of (1 to 1300) Pa at temperatures ≤573 K. In this method, the sample refluxes under an inert buffer gas which transmits the pressure to a pressure transducer operating at ambient temperature. The temperature of the evaporating liquid was determined with a platinum resistance thermometer inserted to a point a few millimeters above the liquid pool. A glass-fiber wick, wrapped tightly around the thermometer, descends into the liquid pool, drawing up boiling liquid by capillary action. This method addresses the twin problems of superheating of the liquid pool and the pressure gradient of approximately 10 Pa mm^–1^ expected in the liquid.^39^ A water-cooled coil above the sensing region of the thermometer was used to condense the rising vapor, establishing an interface between vapor and buffer gas and returning the condensate to the liquid pool. The pressure was measured with a capacitance manometer (BOC-Edwards Barocel, model 622) with an expanded relative uncertainty of Ur (*P*) = 0.0015.*P* (*k* = 2). The uncertainty of the boiling temperature at given pressures is influenced by the boiling regime; uneven or erratic boiling associated with severe superheating may cause fluctuations of several K. In the present work, reasonably smooth and steady boiling was obtained, and the estimated expanded uncertainty of the boiling temperatures was 1 K (*k* = 2).

## 3 Theoretical Correlations

An important key performance indicator in CO_2_ capture processes is the CO_2_ loading (α_i_) capacity of the amine. In this regard, different expressions can be used when an amine present in solution has multiple nitrogen atoms that can potentially react with CO_2_. Table 3 summarizes the CO_2_ loading expressions used in this work. The subscript “*i*” in α_*i*_ expressions indicates the number of nitrogen atoms in any HEIA present that are considered reactive. Moreover, α_0_ measurements are limited in this study to the 0–0.6 loading range.

**Table 3:** Alternative Expressions for CO_2_ Loading in MEA-HEIA-CO_2_ Aqueous Solutions

Expression	Assumption
	HEIA either not present or does not react with CO_2_
	Only one nitrogen atom of HEIA can react with CO_2_
	Both nitrogen atoms of HEIA are capable of reacting with CO_2_

To facilitate the use of the experimental results in process modeling, correlations are generally established. Herein, models from the literature^23,31,40^ are used to validate the data of this work for density, viscosity, and surface tension of loaded and unloaded MEA aqueous solutions. However, the addition of degradation products adds a new degree of freedom in these correlations. For this reason, some models from the literature, originally developed to describe the density and viscosity of carbonated MEA aqueous solutions,^31,40^ are extended to allow the correlation of unloaded and loaded blended amine solutions (e.g., a degradation product). These have been used to correlate the data of this work for density and viscosity of loaded and unloaded MEA-HEIA aqueous solutions. These correlations are described below.

### 3.1 Density Correlations

Weiland et al.^23^ correlation has been used in this work to correlate the experimental density data obtained for aqueous MEA solutions without degradation products. These authors proposed a correlation developed for mixtures with one amine, stating that the density of a solution can be accounted for through a combination of the pure-component and excess molar volumes. Herein, the density ρ is given by its mean molar mass divided by the molar volume *V* of the solution:
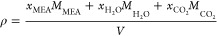
1

In this model, the molar volume of the solution is given by
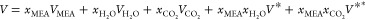
2where *V*_MEA_, *V*_H_2_O_, and V_CO_2__ are the molar volumes of MEA, H_2_O, and CO_2_, respectively, whereas V* and V** are nonideal mixing terms, associated with the MEA-H_2_O and MEA-CO_2_ interactions. The assumption of no reaction or ionization is implicit for the present context, meaning that CO_2_ is not considered as its carbamate or bicarbonate reaction product but as free CO_2_ for mole fraction weighting. In addition, the molar volume of pure amine was correlated using pure component density data^41,42^ and is given as
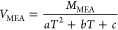
3

The original Weiland’s correlation has been highly discussed in literature and several modifications have been proposed to improve its capacity. Among them, the correlation proposed by Karunarathne et al.^40^ has been used here to represent the measured densities of MEA solutions including degradation products:
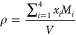
4
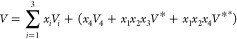
5
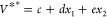
6where *V*_*i*_, *V*, ρ, *M*_*i*_, and *x*_*i*_ are the molar volumes of pure amine and mixture, density of CO_2_ loaded mixture, molar mass, and mole fraction of components in the mixture, respectively. In addition, the subscripts *i* = 1, 2, 3, and 4 refer to HEIA, MEA, H_2_O, and CO_2_, respectively. The molar volume of pure MEA and HEIA were estimated using eq 3. The HEIA density experimental data obtained in this work was used to find the coefficients of the degradation product. Finally, *V*_4_, V*, *c*, *d*, and *e* are temperature-dependent fitting parameters to correlate the dependency of density on temperature:
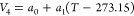
7
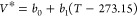
8
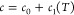
9
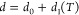
10
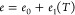
11

It is worth mentioning that, in the original expressions of eqs 7–11 from Karunarathne et al.,^40^ additional higher-order temperature terms are considered, resulting in more complex expressions for the temperature-dependence of the related variables.

### 3.2 Viscosity Correlations

Weiland and co-workers also proposed a correlation to calculate the viscosity of an aqueous amine solution at a given temperature, amine concentration, and CO_2_ loading.^23^
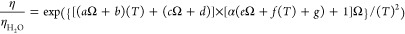
12where η and  are the viscosities of the amine solution and water, respectively (mPa s), Ω is the mass percentage of amine, *T* is the temperature, and α is the CO_2_ loading. According to the literature,^23^ it can be used to calculate MEA solution viscosities up to amine concentrations of 40 mass %, respectively, with CO_2_ loadings up to 0.6 mol of CO_2_/mol of amine for MEA and to a maximum temperature of 398 K.^41−44^ This correlation has also been used in this work to correlate the viscosity of aqueous HEIA solutions, adjusting the parameters of eq 12, particularly *c* and *d*, to the measured data.

In a similar manner as done for the density, Karunarathne et al.^40^ also extended Weiland’s viscosity correlation to mixtures of amines. This extended correlation has been used in this work by considering the degradation products as “additional amines”, as shown in eq 13.
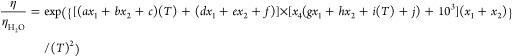
13where η, , *x*_*i*_, and *T* are the viscosity of CO_2_ loaded mixture, viscosity of H_2_O, mole fraction of the compound (*i* = 1, 2, 3, and 4 refer to HEIA, MEA, H_2_O, and CO_2_), and temperature of the liquid mixture. In this expression, it is interesting to note that mole fractions in the mixtures are considered instead of the CO_2_ loading. The coefficients *a* to *g* are the fitting parameters of the expression.

### 3.3 Surface Tension Correlation

Connors and Wright^45^ proposed a correlation for representing surface tensions of binary aqueous–organic solutions that was later extended by Asprion et al.^46^ to ternary mixtures of water + alkanolamine blends. Finally, Jayarathna et al.^47^ introduced a modification to enable the calculation of surface tensions in CO_2_-loaded aqueous alkanolamine binary mixtures. The expression of Asprion et al., adapted to CO_2_-loaded mixtures, is shown in eq 14 and is used in this work to correlate the surface tensions in H_2_O–MEA–CO_2_ mixtures.
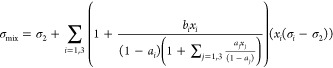
14σ_1_, σ_2_, and σ_3_ correspond to the surface tension of CO_2_, H_2_O, and MEA, respectively, and *a*_1_, *a*_3_, *b*_1_, and *b*_3_ are the fitting parameters of the model. The surface tensions of pure MEA and H_2_O (σ_3_ and σ_2_, respectively) were computed using the DIPPR equation, as presented by Asprion^46^ and described by eq 15:
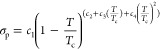
15where *σ*_p_ represents the surface tension of the pure compound, i.e., σ_3_ or σ_2_ in this study, *c*_1_, *c*_2_, *c*_3_, and *c*_4_ are the fitting parameters of the model for each pure compound, and *T*_c_ is its critical temperature.

It is interesting to note that the surface tension of pure CO_2_ is considered as a fitting parameter, as proposed by Jayarathna et al.,^47^ since it does not exist as a liquid above its critical point temperature. Therefore, a linear function of temperature was used to represent the surface tension of CO_2_:
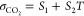
16where *S*_1_ and *S*_2_ are fitting parameters of the expression. The parameters used in this work in eqs 14–16 were previously fitted by Asprion^46^ and Jayarathna et al.^47^

The accuracy of the predictions obtained with the correlations used in this work was evaluated by means of the percentage average absolute relative deviation (AARD), as expressed by eq 17:
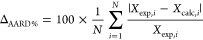
17where *N* is the total number of experimental points, *X*_ex__p,*i*_ and *X*_cal__c,*i*_ are the experimental measurement of a *X* property (i.e. density, viscosity, or surface tension) and its calculated value from the correlation at the same experimental point, *i*. Additionally, the relative deviation (RD) was obtained for each point as
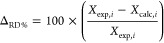
18

## 4 Results and Discussion

Table 4, Table 5, and Table 6 report the density, dynamic viscosity, and surface tension, respectively, of unloaded and CO_2_-loaded aqueous solutions including undegraded 30 mass % MEA and blends containing HEIA in a temperature range from 298.15 to 343.15 K at atmospheric pressure. In these tables, and hereafter, the CO_2_ loading () of the examined solutions are expressed according to the equations presented in Table 3. The main focus of this work is to check the influence of HEIA on the physicochemical properties of the aqueous MEA solution, as HEIA is the most abundant and stable degradation product reported and it does not further react to form other polymeric substances. Additional results for solutions containing MEA with either 4 mass % OZD or 7 mass % HEEDA are presented in Table 7, Table 8, and Table 9.

**Table 4:** Densities ρ of Aqueous Solutions Containing MEA (2), HEIA (3), and CO_2_ (4)^a^

						ρ/(g cm^–3^)							
*x*_2_	*x*_3_	*x*_4_	α_0_	α_1_	α_2_	*T* = 298.15 K	*T* = 303.15 K	*T* = 313.15 K	*T* = 323.15 K	*T* = 333.15 K	*T* = 343.15 K	*T* = 353.15 K	*T* = 363.15 K
0.1128	0	0	0	0	0	1.0106	1.0083	1.0034	0.9981	0.9922	0.9857	–	–
0.1102	0	0.0235	0.213	0	0	1.0634	1.0609	1.0556	1.0502	1.0442	1.038	–	–
0.1091	0	0.0271	0.248	0	0	1.0667	–	1.0589	–	–	1.042	–	–
0.1076	0	0.0459	0.426	0	0	1.1113	1.1089	1.1039	1.0986	1.0927	1.0867	–	–
0.1062	0	0.0527	0.496	0	0	1.1171	–	1.1097	–	1.09841		–	–
0	1	0	0	0	0	–	–	–	–	1.2223	1.2179	1.2043	1.1997
0	0.2722	0	0	0	0	1.1811	1.1776	1.1704	1.1631	1.1557	1.1482	1.1405	–
0	0.1173	0	0	0	0	1.1153	1.1123	1.1061	1.0997	1.0929	1.0858	1.0772	–
0	0.0419	0	0	0	0	1.0527	1.0505	1.0457	1.0404	1.0338	–	–	–
0.1111	0.0130	0	0	0	0	1.0248	1.0224	1.0172	1.0116	1.0055	0.999	–	–
0.1078	0.0127	0.0297	0.275	0.248	0.225	1.0799	–	1.0723	–	–	1.0551	–	–
0.1047	0.0123	0.0576	0.55	0.495	0.450	1.1304	–	1.1229	–	–	–	–	–
0.1101	0.0278	0	0	0	0	1.0405	1.0379	1.0324	1.0265	1.0202	1.0134	–	–
0.1064	0.0266	0.0245	0.233	0.19	0.16	1.0927	1.0896	1.0838	1.0778	1.0719	1.0657	–	–
0.1067	0.0270	0.0304	0.285	0.228	0.19	1.0982	–	1.0902	–	1.0755	1.0724	–	–
0.1038	0.0259	0.0488	0.467	0.37	0.31	1.1369	1.1344	1.129	1.1233	–	–	–	–
0.1036	0.0262	0.0596	0.57	0.456	0.380	1.1369	–	1.1288	–	–	–	–	–

a *x*_*i*_ is the mole fraction of component *i* in aqueous solution, *T* is temperature, and α_*i*_ (*i* = 0, 1, 2) are measures of the CO_2_ loading as determined from the definitions in Table 3. Expanded uncertainties at 95% confidence for MEA aqueous solutions are *U*(*T*) = 0.02 K, *U*(α_0_) = 0.03, *U*(*x*_2_) = 0.0002, and *U*(ρ) = 0.0004 g cm^–3^. Expanded uncertainties at 95% confidence for HEIA aqueous solutions are *U*(*T*) = 0.02 K, *U*(*x*_3_) = 0.0002, and *U*(ρ) = 0.002 g cm^–3^. Expanded uncertainties at 95% confidence for HEIA-MEA blend aqueous solutions are *U*(*T*) = 0.02 K, *U*(α_0_) = *U*(α_1_) = 0.01, *U*(α_2_) = 0.0098, *U*(*x*_2_) = *U*(*x*_3_) = 0.0002, and *U*(ρ) = 0.0002 g cm^–3^. All measurements were performed at a pressure of 0.1 MPa.

**Table 5:** Viscosities μ of Aqueous Solutions Containing MEA (2), HEIA (3), and CO_2_ (4)^a^

						μ/(mPa s)						
*x*_2_	*x*_3_	*x*_4_	α_0_	α_1_	α_2_	298.15 K	303.15 K	313.15 K	323.15 K	333.15 K	343.15 K	353.15 K
0.1128	0	0	0	0	0	2.418	2.089	1.614	1.281	1.043	0.866	–
0.1102	0	0.0235	0.213	0	0	2.988	2.615	2.012	1.618	1.334	1.092	–
0.1091	0	0.0271	0.248	0	0	3.053	–	2.079	–	–	1.138	–
0.1076	0	0.0459	0.426	0	0	3.618	3.139	2.430	1.945	1.583	1.317	–
0.1062	0	0.0527	0.496	0	0	3.674	–	2.531	–	1.652	–	–
0	0.2722	0	0	0	0	21.946	17.339	11.266	7.862	5.687	4.282	3.337
0	0.1173	0	0	0	0	4.443	3.767	2.836	2.182	1.766	1.438	1.182
0	0.0419	0	0	0	0	1.667	1.483	1.175	0.960	0.798	–	–
0.1111	0.0130	0	0	0	0	2.770	2.390	1.814	1.432	1.155	0.950	–
0.1078	0.0127	0.0297	0.275	0.248	0.225	3.765	–	2.382	–	–	1.288	–
0.1047	0.0123	0.0576	0.55	0.495	0.450	4.694	–	2.955	–	–	–	–
0.1101	0.0278	0	0	0	0	3.334	2.825	2.137	1.665	1.338	1.099	–
0.1064	0.0266	0.0245	0.233	0.19	0.16	4.249	3.635	2.758	2.135	1.730	1.424	–
0.1067	0.0270	0.0304	0.285	0.228	0.19	4.389	–	2.748	–	–	1.379	–
0.1038	0.0259	0.0488	0.467	0.37	0.31	5.309	4.576	3.447	2.709	–	–	–
0.1036	0.0262	0.0596	0.57	0.456	0.380	5.336	–	3.546	–	–	–	–

a *x*_i_ is the mole fraction of component *i* in aqueous solution, *T* is temperature, and α_*i*_ (*i* = 0, 1, 2) are measures of the CO_2_ loading as determined from the definitions in Table 3. Expanded uncertainties at 95% confidence are *U*(*T*) = 0.02 K, *U*(α_0_) = 0.03, *U*(*x*_2_) = 0.0002, and *U*(μ) = 0.05 mPa s. Expanded uncertainties at 95% confidence are *U*(*T*) = 0.02 K, *U*(*x*_3_) = 0.0002, and *U*(μ) = 0.05 mPa s. Expanded uncertainties at 95% confidence are *U*(*T*) = 0.02 K, *U*(α_0_) = *U*(α_1_) = 0.01, *U*(α_2_) = 0.0098, *U*(*x*_2_) = *U*(*x*_3_) = 0.0002, and *U*(μ) = 0.01 mPa s. All measurements were performed at a pressure of 0.1 MPa.

**Table 6:** Surface Tensions σ of Aqueous Solutions Containing MEA (2), HEIA (3), and CO_2_ (4)^a^

						σ (mN/m)							
*x*_2_	*x*_3_	*x*_4_	α_0_	α_1_	α_2_	298.15 K	303.15 K	308.15 K	313.15 K	318.15 K	323.15 K	328.15 K	333.15 K
0.1128	0	0	0	0	0	63.570	62.440	61.460	60.570	59.530	59.150	58.180	–
0.1091	0	0.0271	0.248	0	0	67.710	66.645	65.970	65.800	–		–	
0.1062	0	0.0527	0.496	0	0	72.333	71.620	71.720	71.150	–	69.890	68.935	–
0.1111	0.0130	0	0	0	0	63.913	63.165	–	61.223	–	59.180	–	59.454
0.1078	0.0127	0.0297	0.275	0.1078	0.0127	65.960	65.140	64.340	63.846	63.203	62.540	–	–
0.1047	0.0123	0.0576	0.55	0.1047	0.0123	70.813	70.260	69.508	69.153	68.733	68.085	–	–
0.1091	0.0273	0	0	0	0	64.900	64.340	63.773	63.338	62.458	61.705	61.183	60.440
0.1067	0.0270	0.0304	0.285	0.228	0.19	–	67.485	–	66.540	–	64.860	–	–
0.1029	0.0257	0.0586	0.57	0.456	0.380	70.333	70.190	69.542	69.036	–	67.565	–	65.785

a *x*_i_ is the mole fraction of component *i* in aqueous solution, *T* is temperature and α_*i*_ (*i* = 0, 1, 2) are measures of the CO_2_ loading as determined from the definitions in Table 3. Expanded uncertainties at 95% confidence are *U*(*T*) = 0.02 K, *U*(α_0_) = 0.03, *U*(*x*_2_) = 0.0002, and *U*(σ) = 1.2 mN/m. Expanded uncertainties at 95% confidence are *U*(*T*) = 0.02 K, *U*(α_0_) = *U*(α_1_) = 0.01, *U*(α_2_) = 0.0098, *U*(*x*_2_) = *U*(*x*_3_) = 0.0002, and *U*(σ) = 1.2 mN/m.

**Table 7:** Densities ρ of Aqueous Solutions Containing MEA (2), CO_2_ (3), HEEDA (4), and OZD (5)^a^

					ρ/(g cm^–3^)					
*x*_2_	*x*_3_	*x*_4_	*x*_5_	α_0_	298.15 K	313.15 K	328.15 K	333.15 K	338.15 K	343.15 K
0.1072	0	0	8.98 × 10^–3^	0	1.0131	1.0058	–	–	–	0.9881
0.1010	0.0584	0	8.46 × 10^–3^	0.578	1.1250	1.1175	–	1.1065	1.1036	–
0.0959	0	0.0189	0	0	1.0258	1.0182	–	–	–	0.9998
0.0932	0.0280	0.0183	0	0.3	1.0762	1.0679	–	–	1.0533	1.0502
0.0907	0.0544	0.0178	0	0.6	1.1190	1.1113	1.1028	1.0999	1.0968	–

a *x*_i_ is the mole fraction of component *i* in aqueous solution, *T* is temperature, and α_0_ is the measure of the CO_2_ loading (see Table 3). Expanded uncertainties at 95% confidence are *U*(*T*) = 0.02 K, *U*(α_0_) = 0.01, *U*(*x*_2_) = *U*(*x*_3_) = *U*(*x*_4_) = 0.0002, and *U*(ρ) = 0.0003 g cm^–3^. All measurements were performed at a pressure of 0.1 MPa.

**Table 8:** Viscosities μ of Aqueous Solutions Containing MEA (2), CO_2_ (3), HEEDA (4), and OZD (5)^a^

					μ/(mPa s)			
*x*_2_	*x*_3_	*x*_4_	*x*_5_	α_0_	298.15 K	313.15 K	338.15 K	343.15 K
0.1072	0	0	8.98 × 10^–3^	0	2.7471	1.7942	–	0.8666
0.1010	0.0584	0	8.46 × 10^–3^	0.578	4.3457	2.8801	1.6839	–
0.0959	0	0.0189	0	0	2.3394	1.5673	–	0.7667
0.0932	0.0278	0.0183	0	0.3	2.8123	1.9161	–	0.9179
0.0907	0.0544	0.0178	0	0.6	3.4068	2.2972	1.2463	–

a *x*_*i*_ is the mole fraction of component *i* in aqueous solution, *T* is temperature, and α_0_ is the measure of the CO_2_ loading (see Table 3). Expanded uncertainties at 95% confidence are *U*(*T*) = 0.02 K, *U*(α_0_) = 0.01, *U*(*x*_2_) = *U*(*x*_3_) = *U*(*x*_4_) = 0.0002, and *U*(μ) = 0.05 mPa s. All measurements were performed at a pressure of 0.1 MPa.

**Table 9:** Surface Tensions σ of Aqueous Solutions Containing MEA (2), CO_2_ (3), HEEDA (4), and OZD (5)^a^

					σ (mN/m)						
*x*_2_	*x*_3_	*x*_4_	*x*_5_	α_0_	298.15 K	303.15	308.15	313.15 K	318.15 K	323.15 K	328.15 K
0.1072	0	0	8.98 × 10^–3^	0	64.125	63.428	62.667	62.455	61.673	61.098	60.275
0.1010	0.0584	0	8.46 × 10^–3^	0.578	74.585	74.160	73.630	73.060	72.540	71.678	71.185
0.0959	0	0.0189	0	0	64.180	63.596	62.880	61.430	60.468	59.325	57.485
0.0932	0.0278	0.0183	0	0.3	67.240	65.970	65.530	65.020	64.860	64.220	63.380
0.0907	0.0544	0.0178	0	0.6	69.822	69.180	68.665	68.020	67.560	66.900	66.080

a x_*i*_ is the mole fraction of component *i* in aqueous solution, *T* is temperature, and α_0_ is the measure of the CO_2_ loading (see Table 3). Expanded uncertainties at 95% confidence are *U*(*T*) = 0.02 K, *U*(α_0_) = 0.01, *U*(*x*_2_) = *U*(*x*_3_) = *U*(*x*_4_) = 0.0002, and *U*(σ) = 1.2 mN/m.

### 4.1 Density

#### 4.1.1 MEA Solutions

**Figure 2:**
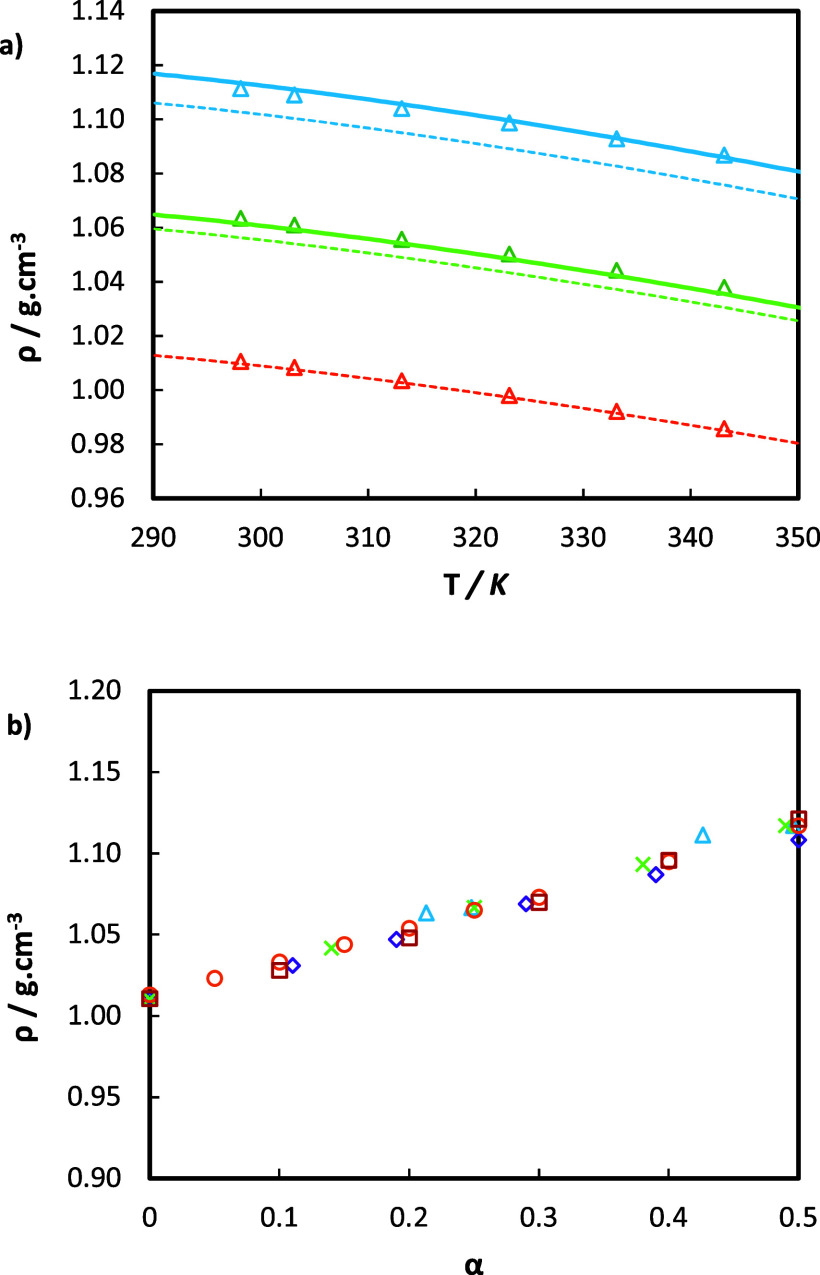
(a) Density, ρ, as a function of temperature *T* for 30 mass % aqueous MEA solutions with different CO_2_ loadings α_0_. This work: orange Δ, α_0_ = 0; green Δ, α_0_ = 0.213; blue Δ, α_0_ = 0.426; - - - and — correspond to the predictions using the Weiland’s model with parameters from ref (23) and with the same parameters but also considering V** fitted in this work (Table 10), respectively. (b) Density as a function of CO_2_ loading at a constant temperature of 298.15 K. Comparison between our data: blue Δ, and literature data: green ×,^48^ purple ◇,^27^ orange ○,^23^ and red □.^50^

The experimentally measured densities of aqueous MEA solutions with and without CO_2_ loading are plotted in Figure 2 and compared with data from the literature^27^.^48^ As can be observed, excellent agreement is found in all cases, even at high CO_2_ loadings. The results reveal the strong impact of the CO_2_ loading on density. For instance, at a temperature of 298.15 K, ρ increases by approximately 10% as α_0_ increases from 0 to 0.43. In contrast, the density decreases by only around 2.5% as the temperature rises from 298.15 to 343.15 K, with α_0_ held constant. In addition, Weiland’s expression has been used to correlate the density measurements (dashed lines in Figure 2 a). The values of the coefficients in eqs 2 and 3 are taken from the original work and are presented in Table 10 for completeness,^23^ whereas the volume of pure water is obtained from the NIST Database.^49^ As observed in Figure 2a), the correlation shows good agreement for the CO_2_-free solution, while some slight underprediction is observed at increasing CO_2_ loadings. In order to obtain a more accurate description of the density of aqueous MEA systems, we have adjusted the parameter V** to accommodate mixing nonidealities associated with MEA-CO_2_ interactions in the corresponding term of eq 2, while retaining the remaining parameters as per ref (23). The new correlated densities are also plotted in Figure 2a) (continuous lines) and have perfect agreement with the data, achieving an overall AARD of 0.06%.

**Table 10:** Parameters Used for Weiland’s Density Correlation (Eqs 2 and 3)^23^

Parameter	Value (30 mass % MEA)	MEA	HEIA
V*	–1.8218 mL mol^–1^		–2.5731 mL mol
*V*_CO2_	0.04747 mL mol^–1^		
*a*		–5.35162 × 10^–7^ g mL^–1^ K^–2^	–4.62536 × 10^–7^ g mL^–1^ K^–2^^a^
*b*		–4.51417 × 10^–4^ g mL^–1^ K^–1^	–9.71437 × 10^–5^ g mL^–1^ K^–1^^a^
*c*		1.19451 g mL^–1^	1.30170 g mL^–1^^a^
V**^a^	–42.0400 mL mol^–1^		
MW		61.08 g mL^–1^	130.15 g mL^–1^

a Adjusted in this work, the other parameters are taken from ref (23).

In Figure 2b), we compare our experimental data for 30 mass % aqueous MEA solutions with other sources analyzing the trend of density as a function of the CO_2_ loading at a constant temperature of 298.15 K. It can be observed that our data fall within the range of the other experimental measurements used for comparison. A similar analysis was performed for a temperature of 313.15 K and is available in Figure S5.

#### 4.1.2 HEIA

**Figure 3:**
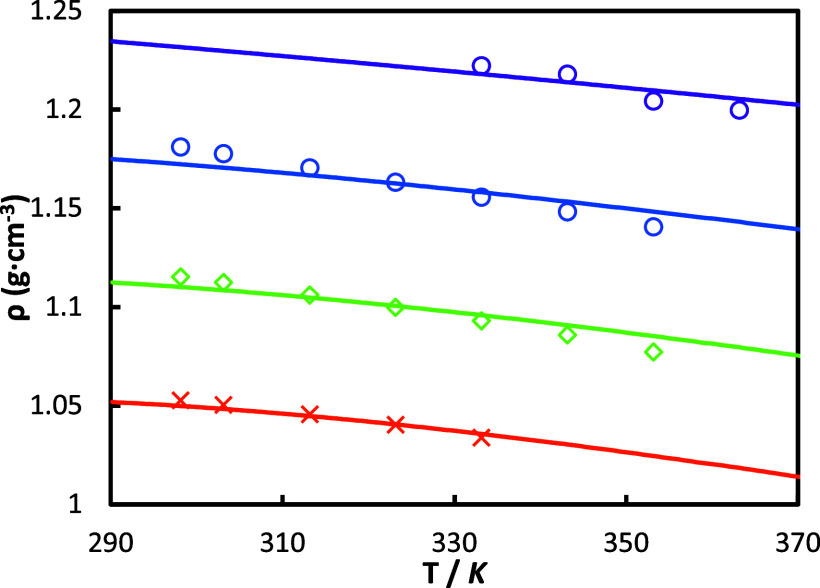
Density ρ of HEIA solution on CO_2_ free basis at different mass fractions: This work: orange ×, w_HEIA_ = 0.24; green ◇, w_HEIA_ = 0.49; blue ○, w_HEIA_ = 0.73; purple ○, w_HEIA_ = 1; and — corresponds to the predictions using the Weiland model with parameters from Table 10.

The density measurements for both pure and aqueous HEIA mixtures are plotted in Figure 3. As observed, the density increases with the HEIA mass fraction and decreases with temperature. The strongest impact is clearly given by the amount of degraded amines in the solution. While a rise from 24 wt % to 100 wt % of HEIA at 298.15 K increases the density by 16%, an increment of temperature from 298.15 to 353.15 K results in a density reduction of 1.8% and 1.9% for the 24 mass % and 100 mass % HEIA mixtures, respectively. Additionally, Figure 3 includes the correlations obtained through Weiland’s model with the parameters adjusted in this work for eqs 2 and 3, revealing a good accuracy, regardless of the composition, with an AARD% of 0.33.

#### 4.1.3 MEA + HEIA Blend Solutions

**Figure 4:**
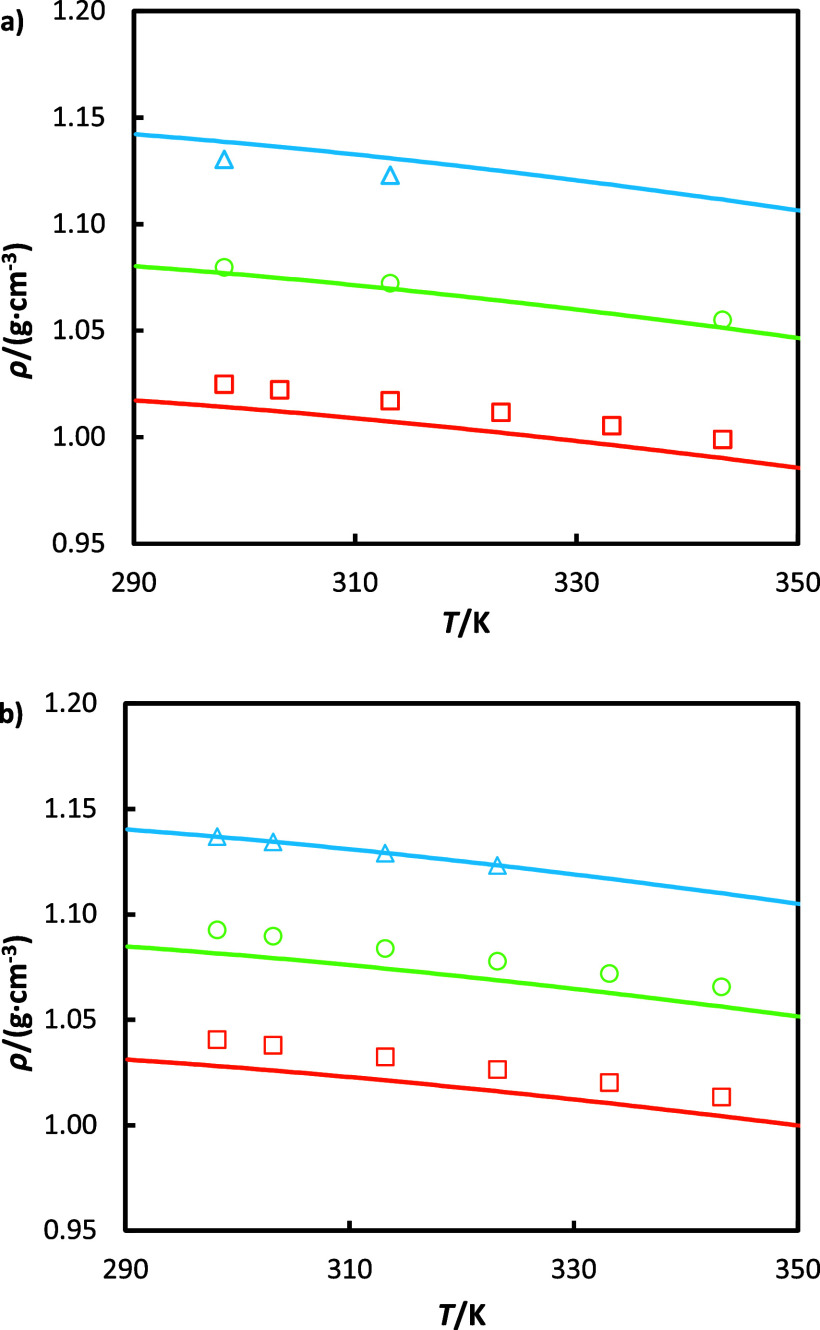
Densities ρ as a function of temperature *T* for aqueous solutions containing (a) 28 mass % MEA and 7% HEIA and (b) 26 mass % MEA and 14% HEIA, respectively, and with different CO_2_ loadings α_0_. This work: (a) orange □, α = 0; green ○, α = 0.275; blue Δ, α = 0.55; (b) orange □, α = 0; green ○, α = 0.23; blue Δ, α = 0.47; and — correspond to the predictions using the Modified Weiland model with parameters from Table 11.

The density measurements of MEA systems are plotted in Figure 4 using different temperature, amine and degraded amine concentration, and CO_2_ loading. The addition of HEIA leads, in all cases, to an increase in density. In unloaded conditions, density increases, on average, by 1.4% and 2.8% for 7 mass % HEIA (Figure 4a) and the 14 mass % HEIA (Figure 4b) systems, respectively, with respect to the benchmark 30 mass % MEA solution.

A very similar pattern is observed for loaded solutions. For half-loaded systems, density increases, on average, by 1.6% and 2.6% for the 7 mass % and 14 mass % HEIA systems, respectively, with respect to the undegraded 30 mass % MEA solution. A similar percentage (1.72% and 2.3%) is found at fully loaded systems. The difference in density between both MEA-HEIA relations is 1.09% and 0.56% for half and full loaded systems, respectively.

As explained in Section 3.1, the new data was correlated by using Weiland’s extension in Karunarathne et al.,^40^ whose fitted parameters are presented in Table 11. As can be seen in Figure 4, it is possible to properly capture the density behavior in the presence of HEIA, given that the AARD % remains low, with a value of 0.73% when considering all the MEA + HEIA mixtures herein tested. Furthermore, Figure S2 provides calculated relative deviations comparing the correlation with each experimental datum of both aqueous HEIA-MEA blends.

**Table 11:** Fitted Parameters for Density Obtained in This Work Using the Modified Weiland Model (Eqs 7–11) and the Experimental Data (Table 4)

Parameter	HEIA-MEA	Parameter	HEIA-MEA
*a*_0_	0	*c*_1_	–5.765 × 10^4^
*a*_1_	2.497 × 10^–1^	*d*_0_	–2.378 × 10^8^
*b*_0_	–3.497 × 10^2^	*d*_1_	2.425 × 10^5^
*b*_1_	–9.001 × 10^–1^	*e*_0_	–2.811 × 10^9^
*c*_0_	–3.770 × 10^5^	*e*_1_	1.792 × 10^5^

### 4.2 Viscosity

#### 4.2.1 MEA Solution

**Figure 5:**
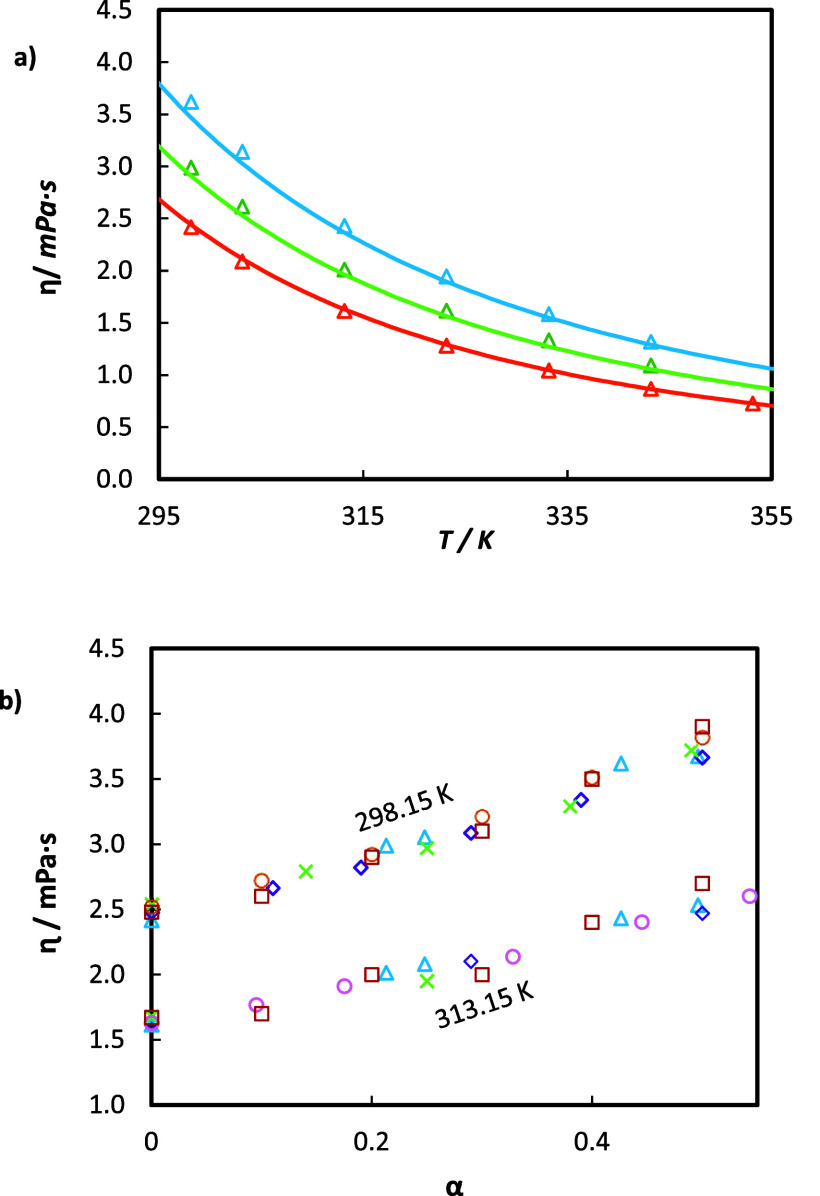
(a) Viscosities η as a function of temperature *T* for 30 mass % aqueous MEA solutions with different CO_2_ loadings α_0_. This work: orange Δ, α_0_ = 0; green Δ, α_0_= 0.213; blue Δ, α_0_ = 0.426; and — correspond to the predictions using the Weiland model with parameters from the literature^23^ (Table 12). (b) Viscosity as a function of CO_2_ loading at constant temperatures of 298.15 K and 313.15 K. Comparison between our data: blue Δ, and literature data: green ×,^48^ purple ◇,^27^ orange ○,^23^ red □,^50^ pink ○.^51^

In Figure 5a, dynamic viscosity measurements for unloaded and loaded aqueous MEA solutions are plotted. The data of the present study are in good agreement with the literature data, with a maximum deviation of 2.0% and 4.8% with Hartono^27^ and Zhang^48^ for the unloaded solution. Regarding loaded solutions, all points follow a clear and coherent tendency; however, a direct comparison cannot be made as the loadings slightly differ from those of experimental sources.

As with density, viscosity decreases with an increase in temperature but increases with the augment of CO_2_ concentration. Quantitatively, a change of α_*i*_ from 0 to 0.43 represents a viscosity change from 2.42 mPa s to 3.62 mPa s (almost a 50% increase), whereas a change of temperature from 298.15 to 343.15 K represents a viscosity decrease of more than a half. These trends are well quantitatively correlated with Weiland’s model (see the curves in Figure 5a), whose coefficients *a* to *g* (see eq 12) are taken from the original work and provided in Table 12 for completeness. The overall AARD is 2.17% and is detailed point by point in Figure S3.

**Table 12:** Parameters Used in This Work the Viscosity Correlation of Weiland (Eq 12)

Parameter	Aqueous MEA 30 mass %^23^	Aqueous HEIA (73, 49, and 24 mass %)^a^
*a*	0	0
*b*	0	0
*c*	21.186	32.486
*d*	2.3730 × 10^3^	1.2409 × 10^3^
*e*	1.015 × 10^–2^	0
*f*	9.300 × 10^–3^	0
*g*	–2.259	0

a Adjusted in this work.

In Figure 5b, our experimental viscosity data for 30 mass % aqueous MEA solutions are compared with the data from the literature to assess measurement accuracy. Data from this work show good agreement with the literature regarding the evolution of viscosity with CO_2_ loading at constant temperatures of 298.15 and 313.15 K.

#### 4.2.2 HEIA

**Figure 6:**
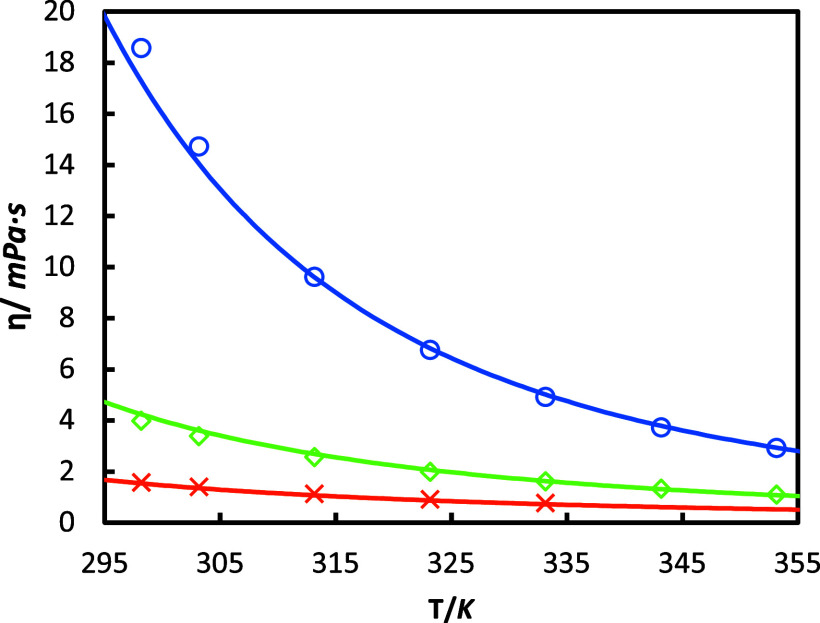
Viscosity η of HEIA aqueous solutions on CO_2_ free basis at different HEIA mass fractions (*w*_2_). This work: orange ×, w_2_ = 0.24; green ◇, w_2_ = 0.49; and blue ○, w_2_ = 0.73, and — correspond to the prediction using the Weiland model with parameters adjusted in this work (Table 12).

The dynamic viscosities of the aqueous solutions of HEIA are illustrated in Figure 6. A highly viscous compound is observed, with a maximum value of 18.6 mPa s at 298.15 K at a mass fraction of 75%, which represents five times greater viscosity than MEA. Even at lower concentrations, HEIA viscosity is still higher than MEA’s. This is a very important finding, given the fact that high viscosities may prevent the absorption of CO_2_.

The impact of water content has, however, an important effect in reducing the viscosity. For example, there is a decrease of 17 mPa s when the HEIA mass fraction changes from 73% to 24% at *T* = 298.15 K. Also, as expected, viscosity reduces rapidly with temperature. Hence, an increase from 298.15 to 353.15 K for HEIA 73% decreases the viscosity by 15.65 mPa s.

Once again, Weiland’s correlations, with the new parameters fitted for HEIA (see Table 12), are compared to the viscosity data in Figure 6, showing an accurate description with an AARD of 3.49% (also refer to RD% in Figure S3).

#### 4.2.3 MEA + HEIA Blend Solutions

**Figure 7:**
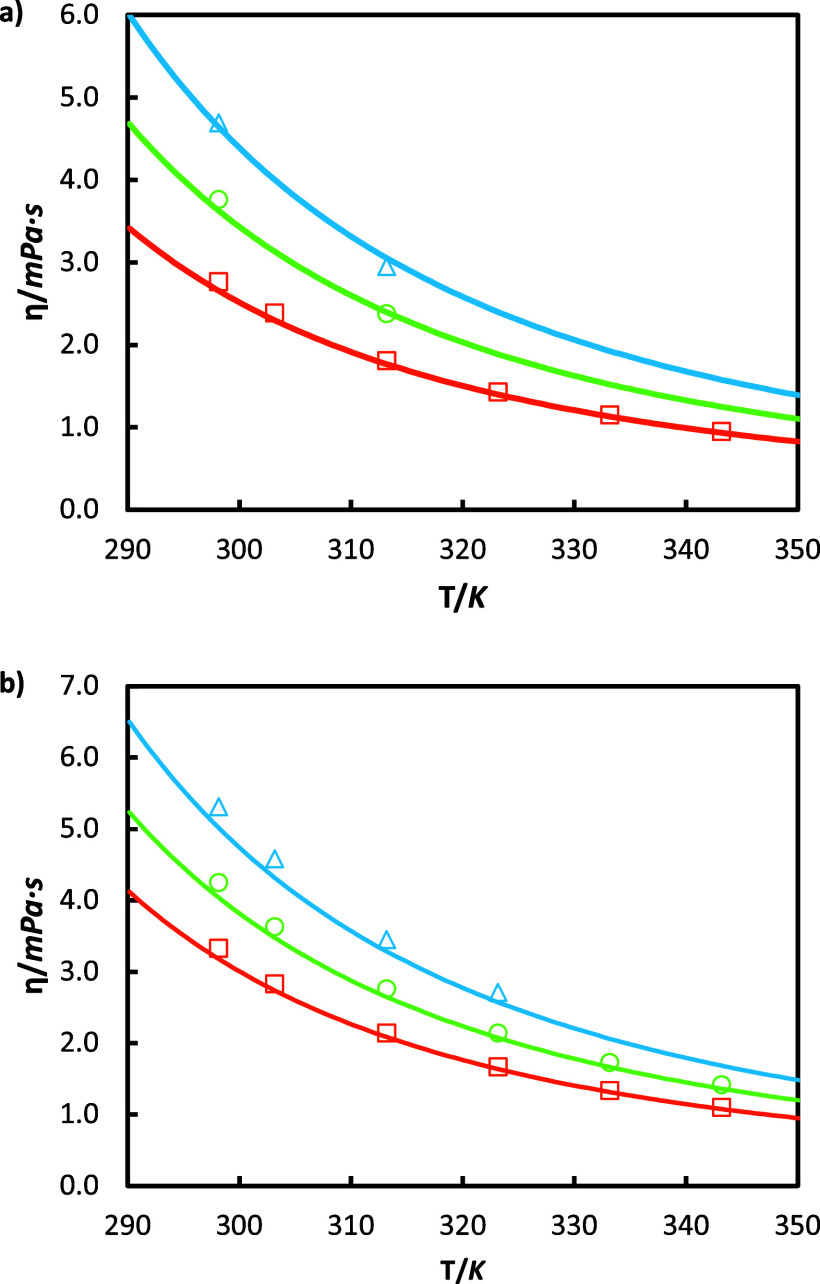
Viscosities η as a function of temperature *T* for aqueous solutions containing: (a) 28 mass % MEA and 7% HEIA and (b) 26 mass % MEA and 14% HEIA, respectively, and with different CO_2_ loadings α_0_. This work: (a) orange □, α_0_ = 0; green ○, α_0_ = 0.275; blue Δ, α_0_ = 0.55; (b) orange □, α_0_ = 0; green ○, α_0_ = 0.23; blue Δ, α_0_ = 0.47; and — correspond to the predictions using the Modified Weiland model with parameters from Table 13.

The viscosity of the solution of degraded amines is now plotted in Figure 7. As for density, dynamic viscosity experiences a clear dependence on temperature, CO_2_ loading, and HEIA content. The viscosity increases with the addition of HEIA and CO_2_ and decreases with temperature. For instance, for unloaded solutions, viscosity increases on average by 12.12% with a concentration of 7 mass % of HEIA and by 31.94% with 14 mass % of HEIA, compared to MEA 30 mass % solution. The difference in viscosity between both unloaded MEA-HEIA blends is notably higher than with density, with an increase of 19.83%.

The change in viscosity is even more pronounced in half and full CO_2_-loaded solutions, with an average increase of 20.84% and 25.64% for a concentration of 7 mass % of HEIA, respectively, and 35% and 43.55% with 14 mass % of HEIA, with regard to the fresh MEA solution.

The new viscosity data were correlated with Karunarathne’s equation (eq 13), whose fitted parameters are displayed in Table 13. This correlation is capable of fitting viscosities for HEIA-MEA blends with acceptable accuracy, as illustrated in Figure 7 and Figure S3, showing an overall AARD of 3.3%.

**Table 13:** Parameters Fitted in This Work for the Modified Weiland Viscosity Correlation (Eq 13) for Aqueous HEIA-MEA Solutions

Parameter	HEIA-MEA	Parameter	HEIA-MEA
*a*	8.555	*F*	108.1
*b*	–0.5859	*G*	–1.017 × 10^5^
*c*	0.2326	*H*	804.8
*d*	713.4	*I*	48.21
*e*	5.241 × 10^3^	*J*	–1.881 × 10^3^

### 4.3 Surface Tension

#### 4.3.1 MEA Solution

**Figure 8:**
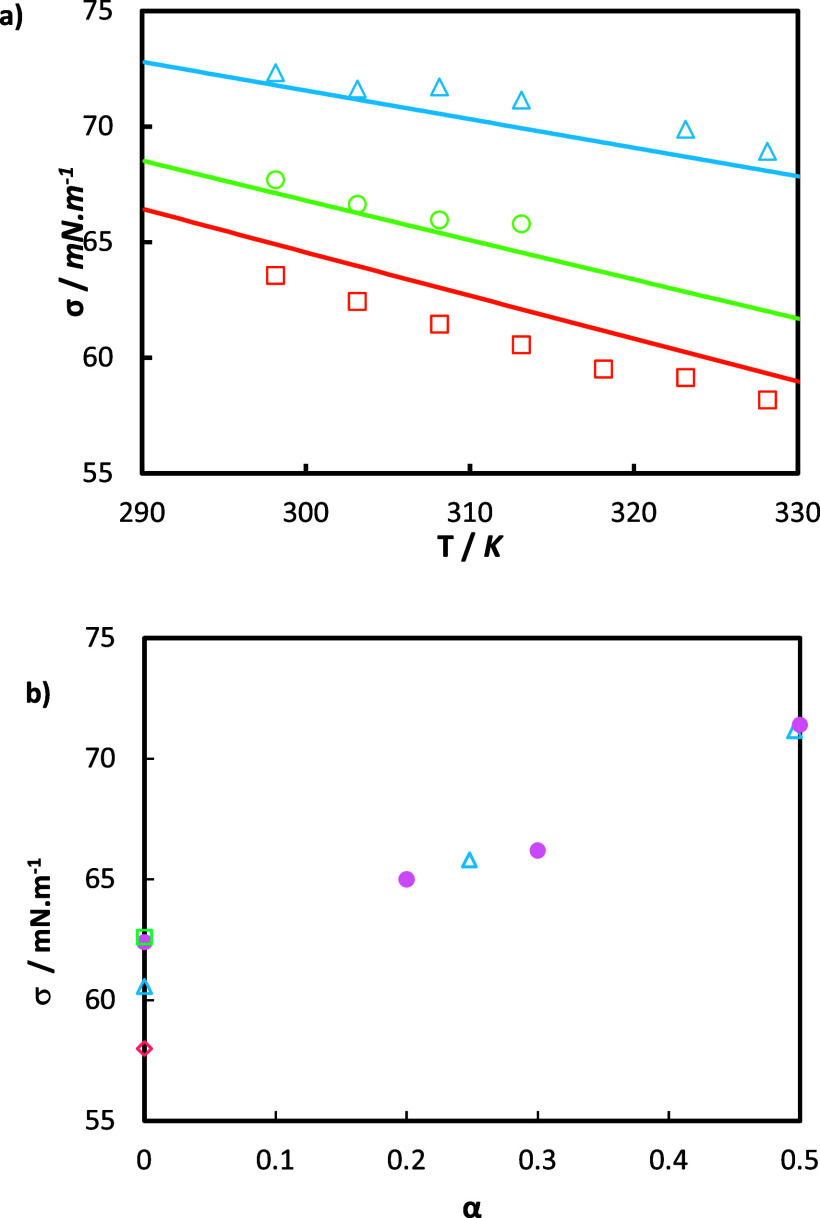
(a) Surface tensions σ as a function of temperature *T* for 30 mass % aqueous MEA solution and solutions formed by dissolving CO_2_ in 30 mass % aqueous MEA with different loadings *α*_0_. This work: orange □, α_0_ = 0; green ○, α_0_ = 0.248; blue Δ, α_0_ = 0.496; and — correspond to the predictions using eqs 14–16 with parameters from refs (46) and (47) (Table 14). (b) Surface tension as a function of CO_2_ loading at a constant temperature of 313.15 K. Comparison between our data: blue Δ, and literature data: orange ◇,^52^ pink ●,^47^ and green □.^31^

Figure 8a illustrates the surface tension of the aqueous solution of MEA at 30 mass % under different CO_2_ loading conditions at temperatures from 298.15 to 333.15 K. The surface tension increases with the rise in CO_2_ loading. This is because the reactivity of aqueous MEA with CO_2_ increases the molecular interactions in the solution. For instance, the surface tension of the 30 mass % MEA increased by 16.54% (±1.8%) when comparing unloaded solutions to fully CO_2_-loaded solutions. Also, as expected, the surface tension decreases with temperature, as the cohesive forces diminish when molecular thermal activity increases.

Additionally, the expressions in eqs 14–16, previously fitted by Asprion^46^ and Jayarathna et al.,^47^ have been used to model the surface tensions and are included in Figure 8 (continuous lines). Figure S4 plots the RD for the correlation for MEA blends. The correlation is capable of fitting surface tensions with acceptable precision showing an accuracy of 0.91% AARD, even though the parameters of the involved expressions (i.e., eqs 14–16) have not been fitted to the experimental data obtained in the present work, but to the data of previous contributions.^46,47^

In Figure 8b, our experimental surface tension data for 30 mass % aqueous MEA solutions are compared with literature data, analyzing their trend with CO_2_ loading at a constant temperature of 303.15 K. Overall, our surface tension data exhibit a consistent increase compared to the data of Jayarathna et al.^47^ (see Table 14). Discrepancies are noticeable at zero CO_2_ loading, particularly with the data of Idris et al.,^52^ which is notably lower. However, our data differs by less than 2% compared to other sources available at zero loading.^47,52^ A similar comparison was conducted at a temperature of 313.15 K and is available in Figure S6.

**Table 14:** Parameters Used in This Work for Surface Tension Correlations (Eqs 14, 15, and 16)

Parameter	Value^47^	Parameter	H_2_O^46^	MEA^46^
*a*_1_	0.09409	*c*_1_	0.18545 N m^–1^	0.09945 N m^–1^
*a*_3_	1.114	*c*_2_	2.717	1.067
*b*_1_	–0.7392	*c*_3_	–3.554	0
*b*_3_	0.1757	*c*_4_	2.047	0
*S*_1_	0.1605 N m^–1^	*T*_c_	647.13 K	614.45 K
*S*_2_	0.0001316 N m^–1^ K^–1^			

#### 4.3.2 MEA + HEIA Blend Solutions

**Figure 9:**
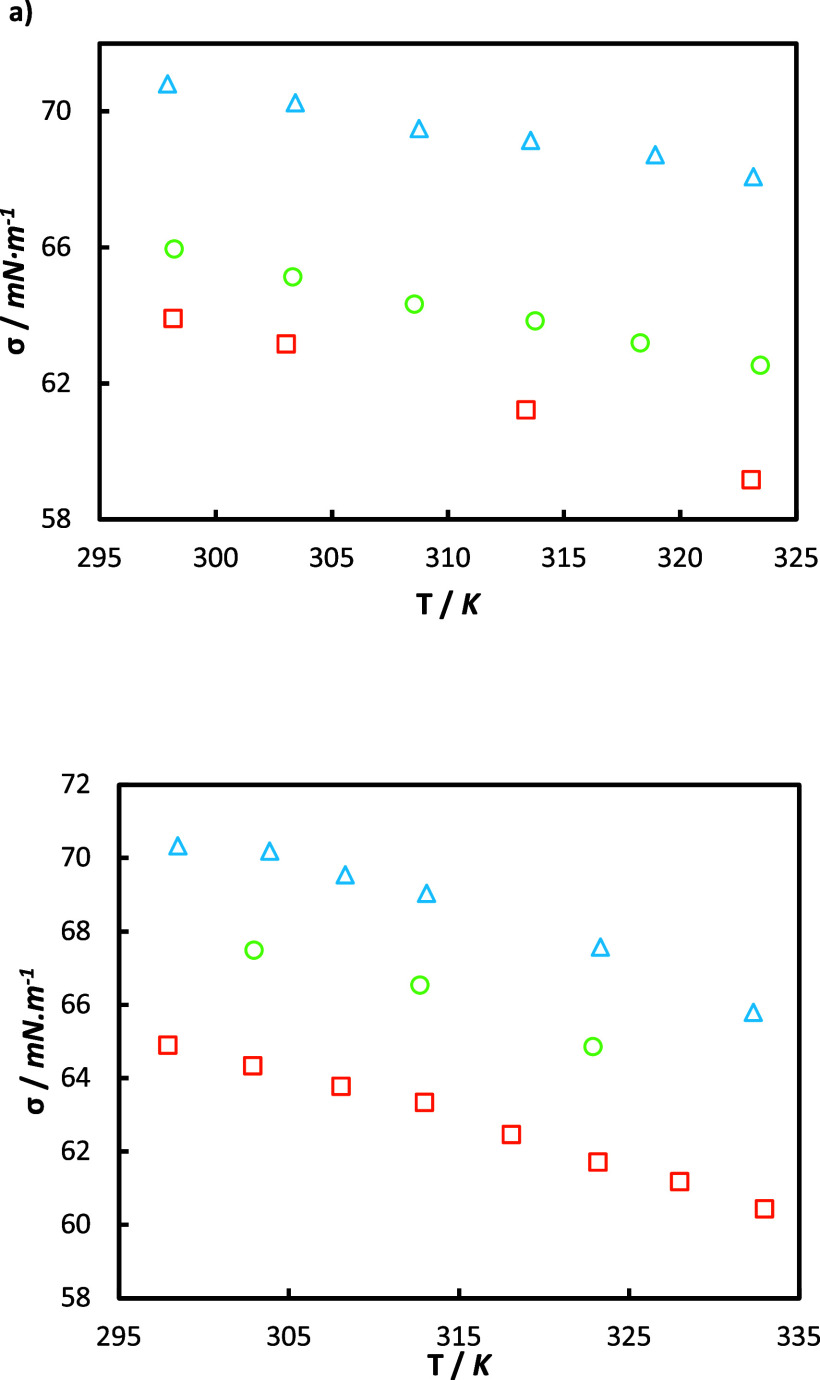
Surface tensions σ as a function of temperature *T* for aqueous solutions containing: (a) 28 mass % MEA and 7% HEIA and (b) 26 mass % MEA and 14% HEIA, respectively, and with different CO_2_ loadings α_0_. This work: (a) orange □, α_0_ = 0; green ○, α_0_ = 0.275; blue Δ, α_0_ = 0.55; (b) orange □, α_0_ = 0; green ○, α_0_ = 0.285; and blue Δ, α_0_ = 0.57.

The surface tension of the HEIA + MEA systems is plotted in Figure 9. A very similar pattern to that observed for the fresh MEA solution is observed, with the surface tension increasing with the CO_2_ content and decreasing with the temperature. However, the behavior of the surface tension with the amount of HEIA requires particular attention, as will be shown in the coming paragraphs.

On a CO_2_-free basis, adding 7 mass % HEIA to the MEA system slightly increases surface tension by 0.71% (±0.4%) compared to the fresh solution. Doubling HEIA concentration to 14 mass % results in a higher rise of 3.98% (±1.0%). However, this trend seems to be affected by the addition of CO_2_ in the system. In fact, at half CO_2_-loaded conditions, the surface tension decreases on average by 1.5% (±1.7%), but increases by 2.3% (±0.34%) for 7 mass % HEIA and 14 mass % HEIA, respectively, compared to the fresh MEA system. Finally, at full CO_2_-loaded conditions, the surface tension decreases on average by 2.5% (±1.7%) and by 2.82% (±0.34%) for 7 mass % HEIA and 14 mass % HEIA, always comparing with the undegraded MEA solution.

The increase of surface tension of the MEA system is higher than that of the HEIA-MEA system when transitioning from unloaded to fully CO_2_-loaded. This occurs because the presence of HEIA limits the increase of the surface tension when the sample is fully loaded with CO_2_. Thus, the more HEIA present, the less the surface tension will increase with CO_2_. This provides a good understanding of the chemical change in the MEA systems at CO_2_-loaded conditions. Surface tension is controlled by the cohesive forces acting on surface molecules. In our experiments, the addition of HEIA to the system is accompanied by a reduction in MEA content, simulating an increase in MEA loss. Consequently, the availability of MEA for reaction is reduced, restricting the formation of the carbamate ions under CO_2_-loaded conditions. The presence of carbamate, along with its cohesive forces, is considered the reason for the elevated surface tension observed under CO_2_-loaded conditions for the 30 mass % MEA system.

### 4.4 Other Blend Solutions

While the major focus of the analysis is given to HEIA as the main degradation product, the densities, surface tensions, and viscosities of CO_2_-loaded and unloaded MEA-HEEDA and MEA-OZD blends were also measured (see Table 7, Table 8, and Table 9).

Similar to MEA-HEIA blends, both HEEDA and OZD increase the density of the fresh aqueous MEA solvent, with HEEDA showing the largest effect. Additionally, the dynamic viscosity of the aqueous MEA system was found to increase in the presence of HEEDA, both under loaded and unloaded conditions, while OZD was observed to only cause a slight increase.

With regard to surface tensions, both HEEDA and OZD increase this property under unloaded conditions compared to aqueous 30 mass % MEA, as done for HEIA. However, under CO_2_-loaded conditions, the addition of HEEDA increases the surface tension compared to the fresh MEA system, while OZD decreases it.

## 5 Conclusions

In this work, an evaluation of the main thermophysical properties of several MEA solutions, including degradation products was carried out. First, the densities, surface tensions, and viscosities of CO_2_ loaded and unloaded aqueous MEA solutions 30 wt % were measured in this work. The model presented by Weiland was used to correlate the density and viscosity data, revealing an AARD of 0.06% and 2.17%, respectively. In addition, the model presented by Jayarathna was used to predict the surface tension of the aqueous MEA solutions, obtaining an AARD of 0.91%.^47^

The influence of the most stable degradation product of MEA, 1-(2-hydroxyethyl)-2-imidazolidinone (HEIA), in MEA aqueous solutions was studied in detail. Due to the lack of experimental data in the literature, the experimental study was divided into two parts. On the one hand, density, and viscosity measurements of HEIA were performed with mass fractions of 100%, 73%, 49%, and 24% over a temperature range from 298.15 to 353.15 K to characterize the degradation product. The density and viscosity of aqueous HEIA solutions were correlated with Weiland’s models, depicting satisfactory accuracy with AARD% values of 0.33% and 3.49%, respectively.

On the other hand, the density, surface tension, and viscosity of unloaded and CO_2_ half-loaded and CO_2_ fully loaded aqueous MEA-HEIA blends (with a mass ratio of MEA of 0.26 and 0.28) at the same temperature range were measured. The presence of HEIA did not inhibit the CO_2_ loading of the MEA systems. Both density and viscosity showed a significant correlation with HEIA concentration and CO_2_ loading. An increase in HEIA and CO_2_ content in the system results in a rise in these two properties.

The modified Weiland’s correlations were adapted in this work for aqueous MEA + HEIA solutions, both CO_2_-loaded and CO_2_-unloaded, and demonstrated to be capable of describing density and viscosity experimental data with 0.73% and 3.30% AARD, respectively, and therefore show a satisfactory representation for engineering calculations.

For surface tension measurements, it was observed that CO_2_ loading increases the surface tension of the mixtures, and HEIA concentration limits the increase in surface tension when the sample is fully loaded with CO_2_. In addition, under CO_2_-loaded conditions, the surface tension of aqueous MEA solvent was higher than for most tested MEA-HEIA blends.

Finally, the densities, surface tensions, and viscosities of CO_2_-loaded and unloaded MEA-HEEDA and MEA-OZD blends were also measured. Overall, these blends were also affected compared to fresh aqueous MEA solutions, with more pronounced effects observed in solutions containing HEEDA than in those containing OZD.

The outcomes obtained in this study, along with the developed models for describing density, viscosity, and surface tension properties, enhance our comprehension of the behavior of degraded MEA solutions, which can contribute to the improvement of the design and operation of the industrial processes of PCC with amine-based technologies.

## Supporting Information Available

HEIA vapor pressure and its equilibrium temperature (Table S1); HEIA vapor pressure in a Clausius-Clapeyron plot (Figure S1); deviations of experimental densities (Figure S2), viscosities (Figure S3), and surface tensions (Figure S4) of this work; and density (Figure S5) and surface tension (Figure S6) as a function of CO_2_ loading at a constant temperature of 313.15 K (PDF)
