# Supplementary material for: Thermophysical Behavior of Carbonated Aqueous Solutions Containing Monoethanolamine and Degradation Products
Source: J Chem Eng Data. 2024 May 9;69(10):3435–49. doi: 10.1021/acs.jced.4c00077 (PMC11603777; doi:10.1021/acs.jced.4c00077)
Supplement: Supplementary file 1 — je4c00077_si_001.pdf [file je4c00077_si_001.pdf]

Supplementary Information

Thermophysical Behaviour of Carbonated Aqueous Solutions Containing Monoethanolamine and Degradation Products

Clàudia Rosa Hernández Narciso[1,2], Cristina G. Martínez [1,2], Brendan O'Connell [2], Sabrina Belén Rodriguez Reartes[3,4], Fèlix Llovell [1,3], J. P. M. Trusler [2], Kyra L. Sedransk Campbell [5]

[1] Department of Chemical Engineering and Materials Science, IQS, Universitat Ramon Llull, Via Augusta 390, 08017 Barcelona, Spain

[2] Department of Chemical Engineering, Imperial College London, South Kensington Campus, London SW7 2AZ, United Kingdom

[3] Department of Chemical Engineering, ETSEQ, Universitat Rovira i Virgili, Av Països Catalans 26, 43007, Tarragona, Spain

[4] Planta Piloto de Ingeniería Química (PLAPIQUI-UNS-CONICET), Camino "La Carrindanga" km 7, Bahía Blanca, 8000, Argentina

[5] Department of Chemical and Biological Engineering, The University of Sheffield, Sheffield S1 4AA, United Kingdom

Properties of pure HEIA

The vapour pressures of HEIA measured in this work in the temperature range of 424.45 K to 442.55 K are shown and plotted against reciprocal temperature in Table S1 and **Error! Reference source not found.** respectively. The low vapour pressures observed indicate that HEIA is counted as a heavy product of low volatility. The enthalpy of vaporisation of HEIA, obtained from experimental data regression, is 139 kJ·mol^{-1}, which is much higher than that of water, MEA and other amines.

Table S1. HEIA vapour pressure in its equilibrium temperature [a].

P (Pa)	Temperature (K)
26.66	424.45
66.66	433.95
133.32	442.55
**ΔH_{vap} (kJ mol^{-1})**	139

[a] Expanded uncertainties at 95% are U (*T*)= 1 and Ur (*P*) = 0.0015·*P*.

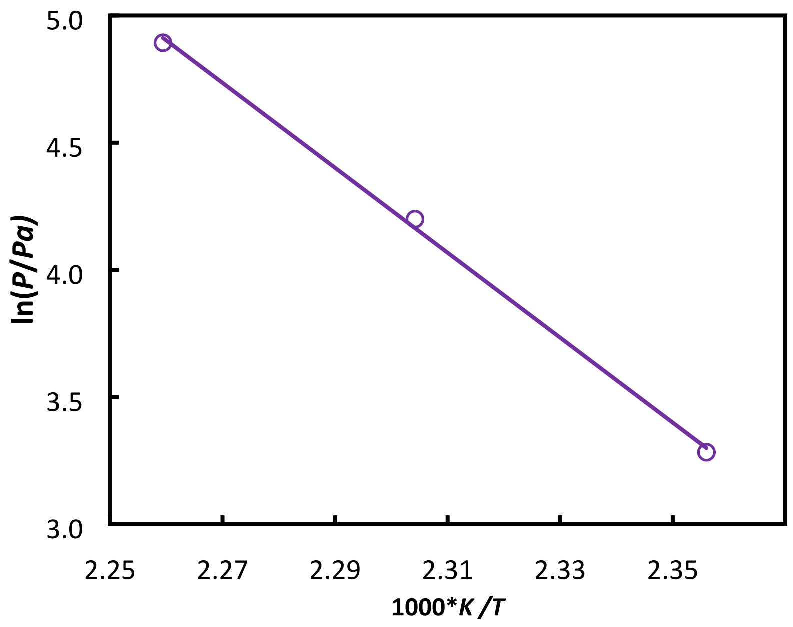

Figure S1. HEIA vapour pressure in a Clausius-Clapeyron plot.

Calculated deviations of correlated properties from experimental measurements

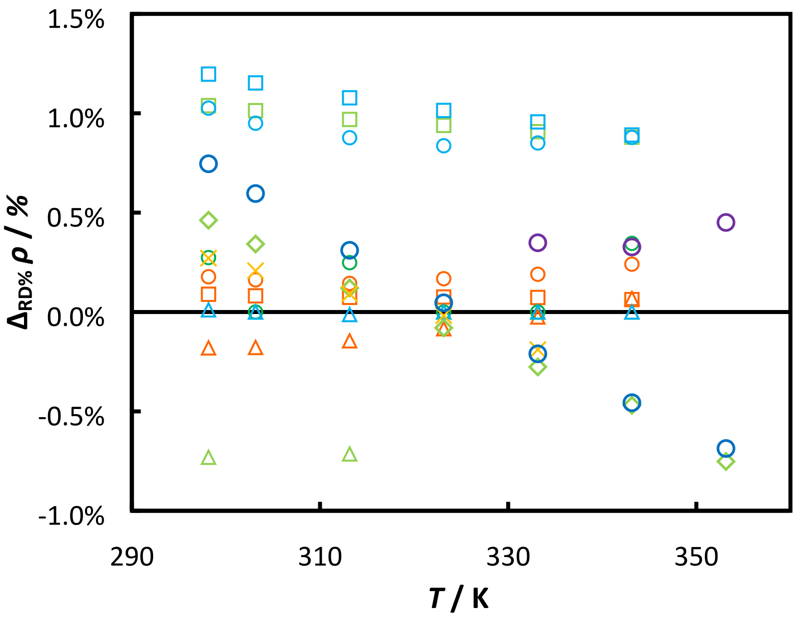

Figure S2. Deviations of experimental densities ρ of this work and calculated based on Weiland and modified Weiland Correlations (Eqs. 1 and 4, parameters from Table 10 and 11) of MEA at 30%wt (orange) and MEA-HEIA blends at [HEIA] 7%wt (green) and 14mass% (light blue): □, unloaded; ○, half loaded; Δ, full loaded; HEIA and aqueous HEIA at: 100%wt (violet) ○; 73%wt (blue) ○; 49%wt (green) ◇; 24%wt (orange) ×.

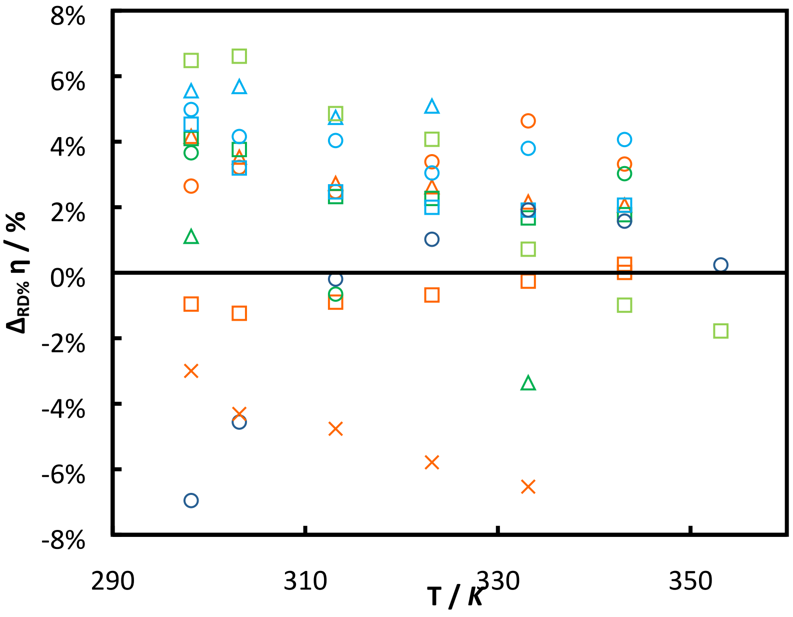

Figure S3. Deviations of experimental viscosities of this work and calculated based on Weiland and modified Weiland's correlations (Eqs. 12 and 13 with parameters from Tables 12 and 13) of MEA at 30%wt (orange); MEA-HEIA blends at [HEIA] 7%wt (green) and 14mass% (light blue): □, unloaded; ○, half loaded; Δ, full loaded; and aqueous HEIA at: 73%wt (blue) ○; 49%wt (green) ◇; 24%wt (orange) ×.

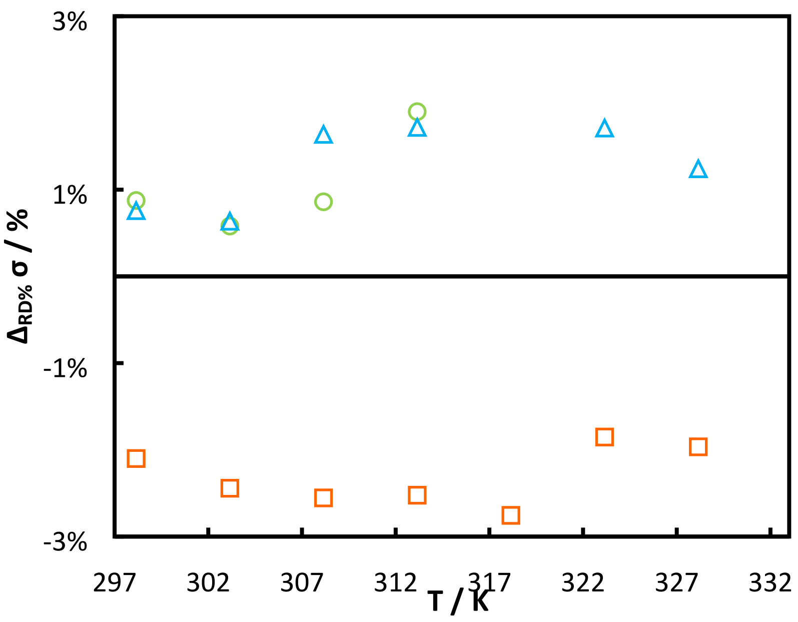

Figure S4. Deviations of experimental surface tensions of this work and calculated based on Eqs. 14-16 with parameters from Table 14 of MEA at 30%wt: □, unloaded; ○, half loaded; Δ, full loaded.

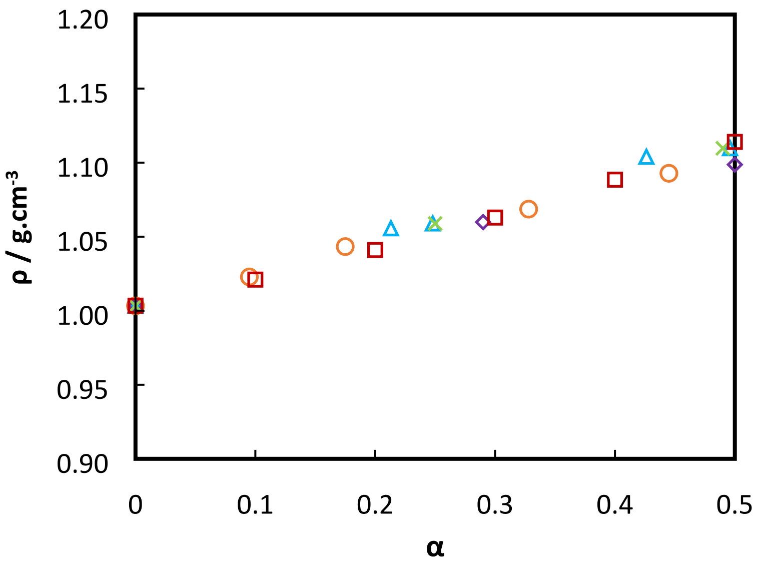

Figure S5. Density as a function of CO_2 loading at constant temperature of 313.15 K. *This work:* Δ*;* ×, [1]*;* ◊, [2]*;* ○ [3]*;* □*, [4].*

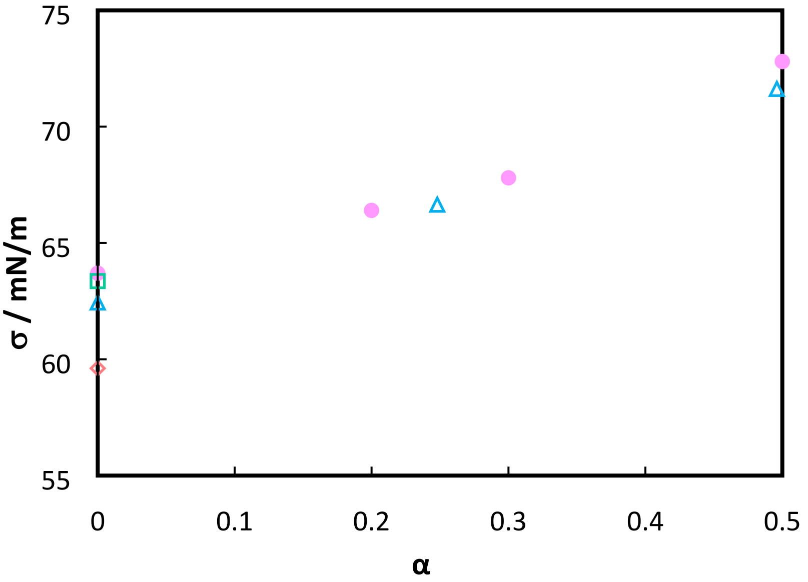

Figure S6. Surface tension as a function of CO_2 loading at constant temperature of 303.15 K. This work: Δ Comparison between our data: Δ, and literature data: ◊, [5]; ●, [6]; □, [7].

References

[1] J. Zhang, P. S. Fennell and J. P. M. Trusler, “Density and Viscosity of Partially Carbonated Aqueous Tertiary Alkanolamine Solutions at Temperatures between (298.15 and 353.15) K.,” Journal of Chemical & Engineering Data, vol. 60, pp. 2392-2399, 2015.

[2] A. Hartono, E. O. Mba and H. F. Svendsen, “Physical properties of partially CO2 loaded aqueous monoethanolamine (MEA),” Journal of Chemical and Engineering Data, vol. 59, p. 1808–1816, 2014.

[3] Karunathane, L.E. Øi, D. A. Eimer. Density, Viscosity and Free Energy of Activation for Viscous Flow of Monoethanol Amine (1) + H2O (2) + CO2 (3) Mixtures”, Fluids 2020, 5, 13.

[4] T. Amundsen, L. Øi and D. Eimer, “Density and Viscosity of Monoethanolamine + Water + Carbon Dioxide from (25 to 80) °C,” J. Chem. Eng. Data, vol. 54, p. 3096–3100, 2009.

[5] Z. Idris, J. Han, S. Jayarathna and D. Eimer, “Surface tension of alkanolamine solutions: An experimentally based review,” Energy Procedia, vol. 114, p. 1828 – 1833, 2017.

[6] S. A. Jayarathna, A. Weerasooriya, S. Dayarathna and D. A. Eimer, “Densities and Surface Tensions of CO2 Loaded Aqueous Monoethanolamine Solutions with r = (0.2 to 0.7) at T = (303.15 to 333.15) K,” J. Chem. Eng. Data, vol. 58, pp. 986-992, 2013.

[7] J. Han, J. Jin, D. Eimer and M. Melaaen, “Density of Water (1) + Monoethanolamine (2) + CO2 (3) from (298.15 to 413.15) K and Surface Tension of Water (1) + Monoethanolamine (2) from (303.15 to 333.15) K,” J. Chem. Eng. Data., vol. 57, p. 1095–1103, 2012.
